# Relationship between Clinical Uterine Findings, Therapy, and Fertility in the Mare

**DOI:** 10.3390/vetsci10040259

**Published:** 2023-03-29

**Authors:** Dragos Scarlet, Eleni Malama, Sophie Fischer, Barbara Knutti, Heinrich Bollwein

**Affiliations:** 1 Clinic of Reproductive Medicine, Vetsuisse Faculty Zurich, Winterthurerstrasse 260, 8057 Zurich, Switzerland; 2 Institute of Veterinary Anatomy, Vetsuisse Faculty Zurich, Winterthurerstrasse 260, 8057 Zurich, Switzerland; 3 Tierarztpraxis KLC, 1562 Corcelles-près-Payerne, Switzerland

**Keywords:** post-breeding endometritis, cervical tone, uterine edema, intrauterine fluid, fertility

## Abstract

**Simple Summary:**

After breeding, mares develop a natural inflammatory reaction of the uterus which then leads to reduced fertility in about 15% of the population. In our study, we analyzed the effects of the inflammatory reaction on fertility of the mares, but also the clinical uterine findings at the time of artificial insemination. Our results show that age and reproductive status of the mare, together with cervical tone, influence mare fertility. Moreover, using cooled-stored semen and performing the artificial insemination after ovulation leads to increased pregnancy rates. Accumulation of intrauterine fluid 12 h after insemination decreased chances of pregnancy, while treatment with oxytocin could slightly improve fertility of affected mares. Taken together, age and reproductive status, together with clinical findings, are useful parameters for assessment of fertility in mares.

**Abstract:**

Persistent breeding-induced endometritis (PBIE) is a major cause of subfertility in horses and the susceptibility is increased by several factors. The aim of this study was to determine the effects of clinical uterine findings and PBIE therapies, respectively, on pregnancy rate in mares. The analysis included records from 220 mares (390 cycles) inseminated at an artificial insemination (AI) center in Switzerland. Gynecological examinations were performed repeatedly before and after AI to determine cervical tone, uterine edema, and intrauterine fluid accumulation. Pregnancy rate was lower (*p* < 0.001) in barren mares compared to mares of other reproductive status. A more flaccid cervix (*p* = 0.009) was observed at the time of ovulation in pregnant cycles, but there was no difference (*p* > 0.05) regarding uterine edema. Intrauterine fluid accumulation reduced pregnancy rate (*p* = 0.002). Oxytocin administration had beneficial effects on pregnancy rate (*p* = 0.015), especially for barren mares, while uterine lavage did not have any effect (*p* > 0.05). The results show that cervical tone and intrauterine fluid accumulation, but not its degree, are useful parameters for assessment of fertility in mares. Oxytocin treatment improved pregnancy rates in mares with PBIE while uterine lavage had a limited effect.

## 1. Introduction

Persistent breeding-induced endometritis (PBIE) is a major cause of subfertility in horses and this phenomenon is reported to occur in up to 25% of mares [1,2]. Upon breeding, the uterus of the mare develops a physiological transient inflammatory reaction in response to the foreign material introduced into the uterine lumen represented by sperm, seminal plasma, and bacteria, with the goal of eliminating it [3]. Fertile mares can successfully complete uterine clearance within 48 h of breeding, thus restoring a suitable environment for the conceptus [4]. In endometritis-susceptible mares, transient breeding-induced inflammation develops into persistent endometritis, which has a negative impact on mares’ fertility [5]. Advanced age, parity of the mare, decreased myometrial contractility, inadequate lymphatic drainage, an insufficiently relaxed cervix, and other unfavorable anatomic and pathophysiologic conditions, such as a large, overstretched uterus that tilts ventrally in relation to the pelvic brim, predispose a mare to PBIE [6,7,8,9].

Several studies demonstrated a reduced pregnancy rate in mares with intrauterine fluid upon insemination, but also in those diagnosed with intrauterine fluid accumulation before insemination [1,5,10,11]. Particularly, older mares are at increased risk of intrauterine fluid accumulation after insemination and consequently susceptible to PBIE due to degenerative changes in the endometrium [9,12]. The endometrium of pluriparous mares exhibits vascular changes leading to reduced uterine blood flow and lymphatic drainage [13]. Moreover, reduced uterine contractility and ventral displacement of the uterus in relation to the pelvic brim have been observed in pluriparous mares and these factors affect uterine clearance [6,14]. In addition, a narrow, fibrotic cervix that opens inadequately during estrus can lead to inadequate uterine drainage and this problem is increasingly encountered in older maiden mares [15,16].

Mares prone to PBIE present decreased fertility and are dependent on therapeutic measures to support uterine clearance for successful insemination. Ecbolic drugs such as oxytocin stimulate myometrial contractions and promote elimination of intrauterine fluid [17]. In PBIE-susceptible mares, oxytocin administration has been shown to improve fertility rates by 7 to 24% compared to untreated controls [5,18]. In addition to oxytocin injections, uterine lavage is another treatment option to physically remove microorganisms, debris, inflammatory cells and mediators, and dead sperm from the uterus [2]. Uterine lavage can be performed starting four hours after insemination to allow enough time for sperm to reach the uterine tubes [19]. An increased conception rate has been demonstrated in mares undergoing uterine lavage 4 to 6 h after insemination compared with mares that received the same treatment 18 to 20 h after insemination [20]. Immunomodulatory agents such as glucocorticoids are also routinely used before breeding to modulate the inflammatory response of the uterus. Both prednisolone and dexamethasone were shown to reduce endometrial inflammation and improve pregnancy rates [21,22].

The occurrence of breeding-induced endometritis and therapy success have been investigated in numerous studies. In most cases, diagnosis has been made based on intrauterine fluid accumulation. The aim of this study was to investigate the relationship between clinical findings, such as cervical tone, uterine edema, intrauterine fluid accumulation, and effects of PBIE therapies on pregnancy rate in mares.

## 2. Materials and Methods

### 2.1. Study Animals

Data were collected from 220 mares (1–5 cycles per mare, 390 cycles in total) presented for artificial insemination (AI) over two consecutive breeding seasons (2013 and 2014) at the KLC Equine Breeding and Insemination Center, Corcelles, Switzerland. A total of 1825 observations were analyzed. The 220 mares included in this study were 12.9 ± 4.4 years old. Based on their reproductive status, 53 mares (24.1%) were registered as maiden, 51 mares (23.2%) as lactating, 58 mares (26.4%) were not bred during the previous season, and 35 (15.9%) were barren, respectively. The remaining 23 mares changed their reproductive status from the first to the second year of the study (e.g., from lactating to barren, from maiden to lactating, etc.).

### 2.2. Gynecological Examination

Mares underwent gynecological examination by transrectal palpation and ultrasonography (7.5 MHz linear probe, MyLab 30 ultrasound machine, Esaote, Cologne, Germany) two to three times daily around the time of insemination. The presence of chronic infectious endometritis was ruled out through endometrial bacteriology and cytology samples which were collected during the first examination of the season from all barren mares and from those presenting intrauterine fluid. The presence of a preovulatory follicle, cervical tone, degree of uterine edema, and intrauterine fluid accumulation were assessed during examination. The cervical tone was assessed by transrectal palpation and classified as follows: 0 = cervix closed, 1 = cervix slightly flaccid and open, 2 = cervix moderately flaccid and open, 3 = cervix markedly flaccid and open. Presence and degree of uterine edema was determined using ultrasonography and classified as follows: 0 = no edema, 1 = low-grade edema, 2 = moderate edema, 3 = marked edema. Amount of intrauterine fluid being visualized in the uterus was classified as: 0 = no fluid, 1 = fluid < 2 cm, 2 = fluid > 2 cm, as previously described [23].

In all mares, ovulation was induced with 3000 IU hCG i.v. upon detecting a preovulatory follicle ≥35 mm and uterine edema ≥2, followed by gynecological examinations every 12 h. In mares bred with cooled-stored semen, AI was performed 24–36 h after hCG application, depending on the time when semen was delivered. In few mares, an ovulating preovulatory follicle was detected at the time of examination, thus the AI was done shortly after ovulation had started. In mares bred with frozen-thawed semen, AI was done 36 h after induction of ovulation. In case ovulation had occurred before, AI was done at the first examination when a collapsed ovulatory follicle was detected. Out of the 390 cycles included in the study, cooled-stored semen was used in 74 cycles (19% of cycles), while frozen-thawed semen was used in 316 cycles (81% of cycles). In 219 out of 390 cycles (56%) AI was performed prior to ovulation, while in 171 out of 390 cycles (44%) AI took place after the ovulation, respectively. Pregnancy was confirmed by the presence of a conceptus during ultrasonographic examination at 16.6 ± 2.6 days after ovulation.

### 2.3. Treatment Regime

Mares were examined 12 h after insemination and, in case intrauterine fluid <2 cm was diagnosed, treatment with 20 IU oxytocin intravenously was applied two times, 12 h apart, during the first 24 h after insemination, as previously described [20]. In 10% of the mares with intrauterine fluid <2 cm, where an extra examination was performed, a third shot of oxytocin was given within 24 h. Whenever intrauterine fluid ≥2 cm was diagnosed, uterine lavage was performed with 0.9% saline solution using a catheter (outer diameter 12 mm, inner diameter 8 mm, length 80 cm) equipped with a balloon (Bivona, Provet AG, Lyssach, Switzerland) additionally to oxytocin treatment. Uterine lavage was repeated until the recovered fluid no longer showed turbidity.

### 2.4. Analysis of the Data and Statistical Evaluation

Data included in the study were collected from 48 h before until 48 h after AI. Foal heat cycles, mares examined less than twice before ovulation, and those for which no time of ovulation was documented were not included in the analysis. In addition, only cycles with ovulation occurring within 48 h after hCG injection and where semen with progressive motility >40% was used were included in the analysis.

Statistical analysis was performed using the R version 4.1.4 language for statistical computing (The R Development Core Team, 2021). The mean and SD were used as dispersion measures of numeric variables. Categorical variables were summarized in form of contingency and frequency tables. In total, 1825 repeated measures were made for 220 mares (105, 72, 32, eight, and three mares examined over one, two, three, four, and five consecutive cycles, respectively). The Wilcoxon rank sum test, a rank-based nonparametric test, was used to determine if there are statistically significant differences (at 0.05 significance level) of a continuous or ordinal variable in pregnant vs. non-pregnant animals. 

To assess the effect of cervical tone, uterine edema, intrauterine fluid, oxytocin treatment, and uterine lavage, as well as their interaction with the age of the mare and the time of assessment/application prior to/after AI or ovulation on the occurrence of pregnancy, a generalized linear mixed model was applied. Binomial generalized linear mixed models (GLMM) were applied for modeling the binary response of pregnancy outcome (occurrence vs. no occurrence of pregnancy for each single cycle) for clustered measures of the fixed predictors cervical tone, uterine edema, intrauterine fluid, oxytocin treatment, and uterine lavage. The interaction terms of the above-mentioned predictors with the 3rd-order polynomial of age, the reproductive status of the mare, the time of examination in relationship to ovulation or AI, as well as the type of semen and the time of AI were also incorporated in the model as fixed effects. The intercept of the model was allowed to vary for each level of the random effects of mare and mare’s cycle. The exponentiated estimates of the regression coefficients (i.e., odds ratios), the corresponding 95% confidence intervals (CI), and *p* values were reported for the fixed effect of each predictor on the pregnancy outcome, using 0.05 as threshold for the significance level. The predicted probability values were computed to express the likelihood of pregnancy at cycle level. Only complete sets of observations, available for 209 mares (1755 observations in total with 98, 70, 30, eight, and three mares examined over one, two, three, four, and five consecutive cycles, respectively), were used as input for GLMM.

The probability of different cervix tone or uterine edema grades to occur with progressing age was assessed by fitting cumulative link mixed models. The random effect of mare’s cycle, nested under the random effect of mare, was also incorporated in the model. The sensitivity of the applied model was 90.3%, whereas the specificity was 90.9%. Model parameters (log-odds) were reported using a 0.05 significance level as threshold.

## 3. Results

### 3.1. Mare Fertility in Relation to Age and Reproductive Status

**Figure 1:**
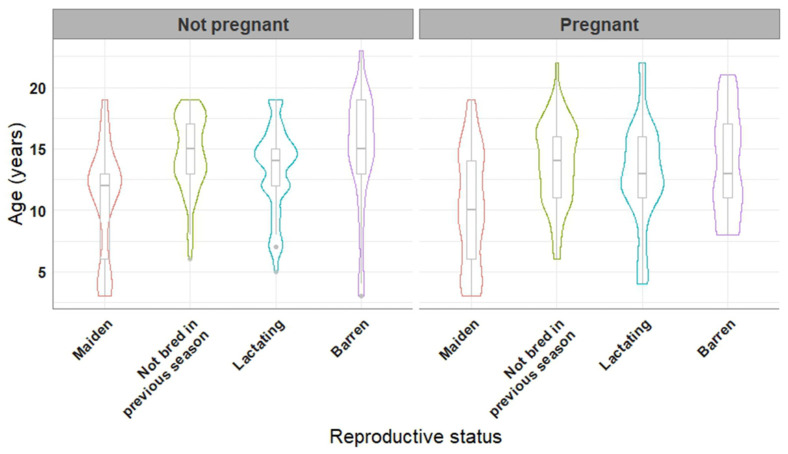
Violin and boxplots of the age of 220 mares in relation to their reproductive status. Mares that did not get pregnant vs. mares that got pregnant at least once during the two-year study are presented in the left and right panels of the figure, respectively.

Age of the mares which became pregnant was lower than that of mares which failed to become pregnant (12.6 ± 4.5 years vs. 13.2 ± 4.4 years, *p* = 0.015). The pregnancy rate differed (*p* < 0.001) between mares of diverse reproductive status, with 52.1%, 57.5%, 58.7%, and 21.9% of the examined cycles resulting in pregnancy in maiden, lactating, mares not bred in the previous season, and barren mares, respectively. The age distribution of the mares in relation to their reproductive status and to the pregnancy outcome is illustrated in Figure 1.

### 3.2. Mare Fertility in Relation to Semen Type and Time of Insemination

**Figure 2:**
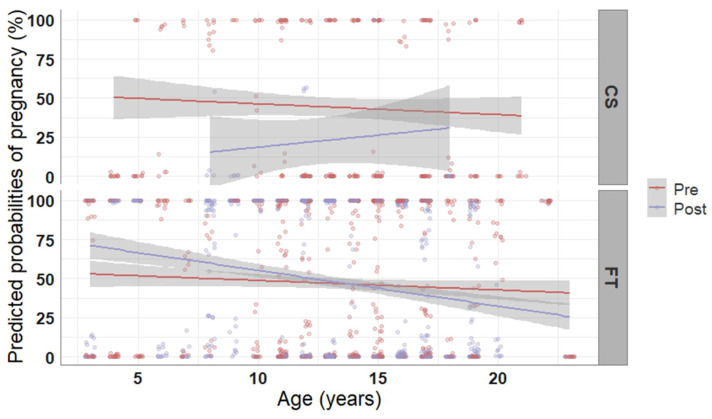
Predicted probabilities of pregnancy plotted in relation to the age of the mare, the time of artificial insemination, and the type of semen used. Pregnancy probabilities were computed based on 1755 observations of 210 mares using a generalized linear model. The mean probability of pregnancy with progressing age is represented with solid lines; the grey-shaded areas reflect the 95% confidence intervals of the mean probability line. CS, cooled-stored semen; FT, frozen-thawed sperm; Pre, prior to ovulation; Post, after ovulation.

Semen type (frozen-thawed vs. cooled-stored) and time of AI (prior to vs. after ovulation) both affected pregnancy rate. Particularly, mares inseminated with frozen-thawed semen had less chances (−6.32 ± 1.11 log-odds, *p* < 0.001) of becoming pregnant compared to those inseminated with cooled-stored semen (43.2% vs. 48.7%). Time of AI also affected the likelihood of pregnancy. Mares bred after ovulation had higher odds to become pregnant compared to mares bred prior to ovulation (2.97 ± 0.60 log-odds, *p* < 0.001). The observed pregnancy rate was 48.5% in case of AI after ovulation and 47.0% if insemination was carried out prior to ovulation (83/171 vs. 103/219 examined cycles, respectively). However, this pattern was dependent on the type of semen used for AI and the age of the mare, as demonstrated in Figure 2. In general, pregnancy rate decreased as age of the mares increased, besides in the few cases of mares bred with cooled-stored semen post ovulation. In mares bred with frozen-thawed semen, performing AI post ovulation increased pregnancy rate in mares up to 12 years of age but decreased it in older mares, whereas an AI prior to ovulation led to constant pregnancy rate, independent of the age of the mare. For cooled-stored semen the pregnancy rate was 44.8% in case of AI prior to ovulation and it decreased to 25.0% in case of AI after ovulation, respectively. The opposite was seen for frozen-thawed semen, where AI prior to ovulation resulted in 47.7% pregnancy rate, whereas AI after ovulation resulted in 49.7% pregnancy rate.

### 3.3. Associations between Clinical Uterine Findings and Mare Fertility

**Figure 3:**
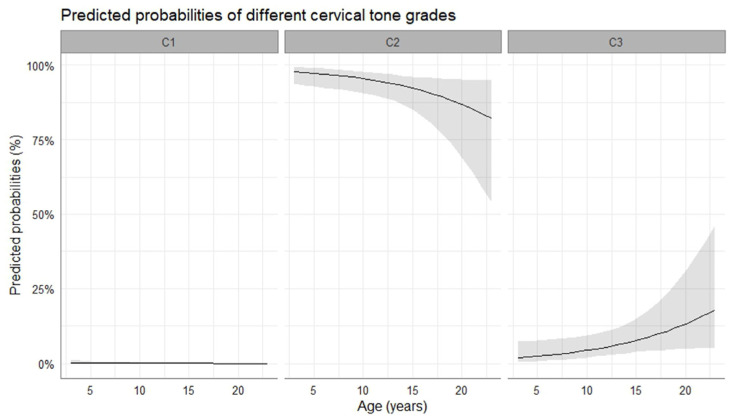
Mean probability (solid line) and the respective 95% confidence intervals (grey-shaded area) for the occurrence of specific cervical tone with progressing age of the mare. C1, cervix slightly flaccid and open; C2, cervix moderately flaccid and open; C3, cervix markedly flaccid and open.

At the time of ovulation, most mares were diagnosed with a cervical tone grade 2 or 3. The probability of cervix tone 1 to be observed was close to zero, while cervix tone 0 was not present. It was more likely to observe a grade 3 (i.e., markedly flaccid and open cervix) than a grade 2 (i.e., moderate flaccid and open cervix) with advancing age of the mare (4.28 ± 0.87 log-odds, *p* < 0.001). The predicted probabilities of different cervical tones to occur in relation to the age of the mare are presented in Figure 3. Moreover, a more flaccid cervix was observed at the time of ovulation in pregnant cycles compared to non-pregnant cycles (2.3 ± 0.5 vs. 2.2 ± 0.6, *p* = 0.009). Furthermore, cervical tone appeared to change over time prior to ovulation, as the cervix became more flaccid with impending ovulation.

**Figure 4:**
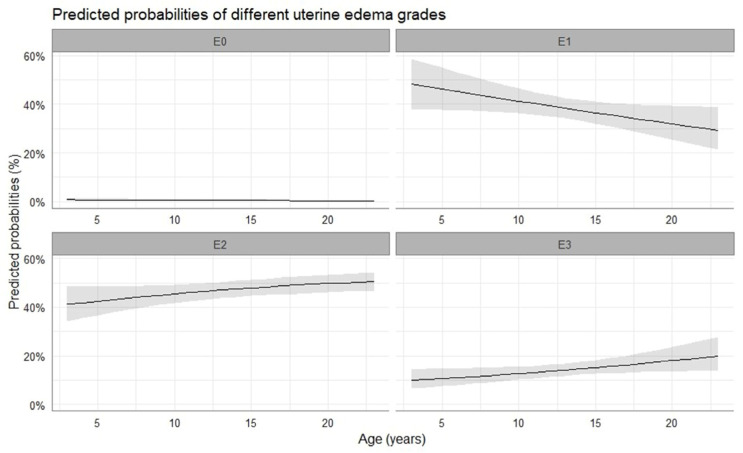
Mean probability (solid line) and the respective 95% confidence intervals (grey-shaded area) for the occurrence of uterine edema with progressing age of the mare. E0, no edema; E1, low-grade edema; E2, moderate edema; E3, marked edema.

Most mares were diagnosed with a uterine edema grade 1, 2, or 3, while the probability of uterine edema grade 0 to be observed was close to zero. Uterine edema decreased towards the time of ovulation and thereafter increased again, but it did not differ at the time of ovulation between pregnant and non-pregnant cycles (*p* > 0.05). Similar to cervical tone, the grade of uterine edema observed at the time of ovulation was affected by the age of the mares (*p* < 0.05). There was a shift from moderate (grade 2) to marked (grade 3) uterine edema at the time of ovulation with increasing age of the mare (2.34 ± 0.28 log-odds, *p* < 0.001), while chances for low-grade (grade 1) uterine edema to be observed decreased (0.04 ± 0.02 log-odds, *p* = 0.038). The predicted probabilities of different uterine edema grades to occur in relation to the age of the mare are presented in Figure 4.

### 3.4. Effect of the Treatment Regime on Mare Fertility

Twelve hours after insemination, in 51.8% of cycles no intrauterine fluid was detectable, whereas in 32.2% of cycles fluid accumulation <2 cm and in 16.0% of cycles fluid accumulation >2 cm was detected, respectively. Mares that had not been bred during the previous season (60.9%, 56/92 cycles) and barren mares (61.5%, 59/96 cycles) accumulated intrauterine fluid more frequently than maiden (51.0%, 49/96 cycles) and lactating mares (23.6%, 25/106 cycles). Pregnancy rate was 43.9% (83/189) for cycles with intrauterine fluid accumulation vs. 50.7% (102/201) for cycles without fluid. The pregnancy probabilities were affected by the interaction of intrauterine fluid accumulation and the reproductive status of the mare. Particularly, lactating mares and mares not bred during the previous season with no intrauterine fluid were more likely (*p* < 0.05) to become pregnant (57.2 ± 2.2% and 62.0 ± 2.6% probability, respectively) compared to mares of the same reproductive status with intrauterine fluid <2 cm (50.0 ± 8.8% and 44.5 ± 6.1% probability, respectively). Oxytocin treatment had beneficial effects on probability of pregnancy (11.97 ± 4.92 log-odds, *p* = 0.015) based on the results of logistic regression. This effect was mainly observed in barren mares which had 20.7 ± 1.9% probability of pregnancy in the absence of intrauterine fluid and 40.1 ± 5.2% pregnancy rate when intrauterine fluid <2 cm was present and treated with oxytocin alone. Overall, chances for pregnancy were lower in cycles with intrauterine fluid <2 cm compared to cycles without intrauterine fluid (−14.33 ± 4.54 log-odds, *p* = 0.002).

Regarding mare fertility in relation to uterine lavage and the reproductive status of the mare, lactating mares required less uterine lavage than mares that were not bred the previous season, maiden, and barren mares (5.7% vs. 17.4%, 20.8% and 32.3% of the examined cycles, respectively). Pregnancy rate was 30.1% (22/73) for cycles where intrauterine fluid >2 cm was diagnosed and 51.6% (163/316) for the rest of the cycles. All but one mare that required uterine lavage also received oxytocin therapy at the same time.

## 4. Discussion

Herein we investigated the relationship between clinical uterine findings around the time of ovulation, PBIE therapy, and fertility in mares. Older mares showed a lower pregnancy rate than younger mares. An explanation for this is found in the close relationship between age, endometrial degeneration, and intrauterine fluid accumulation after insemination [24]. Older mares show lower uterine contractility and delayed fixation of embryonic vesicle, but also fibrotic changes and less dense endometrial glands compared to younger mares [14]. These age-related changes increase susceptibility for PBIE and were associated with altered placental development and higher pregnancy losses [25,26]. In addition to age, reproductive status also affected the fertility of mares. Barren mares were less likely to become pregnant (21.9%) compared to mares with other reproductive status (52.1% to 58.7%). Again, this observation is consistent with results of other studies in which barren mares had significantly lower pregnancy rates and suffered more pregnancy losses compared to maiden and lactating mares, respectively [24,27,28]. A close association between the degree of endometrosis and reproductive status was observed in an analysis of 2500 endometrial biopsies, with endometrosis being diagnosed in more than 70% of the barren mares [29]. Since degenerative changes lead to impaired uterine function, this may explain reduced fertility in barren mares.

Under estrogen influence, prominent endometrial edema is physiologic and presents on ultrasound as a typical structure. Uterine edema reaches its maximum expression four days before ovulation and then decreases until the time of ovulation [30]. This phenomenon was also reflected in this study. Degree of uterine edema did not influence pregnancy rate in our study. Most mares presented a low- or moderate-degree uterine edema at the time of ovulation. Previously, a 10–14% decrease in pregnancy rate was observed in mares with marked uterine edema at the time of ovulation [31]. One explanation for this could be endometrial vascular degeneration leading to decreased lymphatic drainage and, in extreme cases, also intrauterine fluid accumulation. Uterine lymphatics aid in the elimination of fluid contents from the uterine lumen and allow for the reduction of uterine edema [32]. We also observed a shift towards marked uterine edema in older mares in our study. Since endometrosis and angiopathies are age-associated diseases [33], increased endometrial edema is to be expected in older mares.

Regarding cervical tone, mares which became pregnant presented a more relaxed cervix at the time of ovulation than mares with unsuccessful insemination. This confirms the important role of the cervix in uterine clearance [8,15,34]. Cervical tone reduces under the influence of high estrogen levels during estrus to allow sperm to enter the uterus. Furthermore, an open cervix facilitates the elimination of inflammatory products and fluid from the uterus following insemination. If drainage is not working due to inadequate opening of the cervix, this results in accumulation of intrauterine fluid [15]. In our study, the chances for a markedly flaccid and open cervix to be diagnosed at the time of ovulation increased with advancing age of the mare. This is an interesting result, as fibrosis and loss of cervical elasticity were previously attributed to foaling and manipulation of the cervix; however, inadequate cervical patency is also observed in maiden mares, especially the older ones [8]. The mares included in this study were evenly distributed according to age and reproductive status; thus, age had a direct effect on cervical tone. Based on reproductive status, lactating mares had the least intrauterine fluid accumulation and the second highest pregnancy rate (57.2%) among groups. Several studies have already shown that lactating mares have similar pregnancy rates to maiden mares [1,24,28,32]. At the same time, lactating mares seem to be less susceptible to PBIE, especially if being bred during the first 60 days after foaling [24]. The reason behind this is still unclear. One can speculate that this is an effect of the regular suckling of the foal leading to increased uterine contractility and consequently to better clearance. Although oxytocin release in response to suckling seems to be limited [35], a clear increase in uterine activity can be induced by administration of exogenous oxytocin [36].

Twelve hours after insemination, intrauterine fluid accumulation was present in almost half of the analyzed cycles. Compared with the study by Zent et al. [32] in which intrauterine fluid was observed in 15.6% of cycles after insemination, the incidence of intrauterine fluid in the present study (48.2%) appears to be high. However, the latter authors diagnosed intrauterine fluid 14 to 30 h after insemination; thus, the uterine fluid could have been physiologically cleared by then. Furthermore, all mares received an intrauterine infusion with antibiotics immediately after breeding in the respective study [32], which could reduce the incidence of intrauterine fluid, whereas in the current study, mares did not receive any therapy until after intrauterine fluid was diagnosed. Other studies report 25–31% of the mares within a population requiring therapy [1,27]. These results are comparable with our findings, considering that herein also fluid accumulation <2 cm was taken into account and that the aforementioned studies had a lower percentage of barren mares included in the analysis. The interaction between intrauterine fluid accumulation and the reproductive status of the mare influenced the pregnancy rate per-cycle. Lactating mares and mares not bred during the previous season were more likely to become pregnant in the absence of intrauterine fluid and this is in accordance with previous studies [1]. However, pregnancy rate of barren mares significantly improved in the presence of intrauterine fluid <2 cm, most likely because of the beneficial effects of administrated oxytocin [17]. Overall, chances for pregnancy per-cycle were reduced in case of intrauterine fluid accumulation after insemination in our study.

Fertility of the mares was influenced by the type of semen used and by the time of artificial insemination. Mares inseminated with cooled-stored semen had a higher pregnancy rate per-cycle than mares inseminated with frozen-thawed semen. A stronger inflammatory response of the endometrium and consequently greater fluid accumulation have been described after insemination with frozen-thawed semen compared to cooled-stored semen [37,38]. Thus, the lower pregnancy rates obtained with frozen-thawed semen in this study can be due to damage suffered by sperm during the freezing and thawing process [39] but also due to higher frequency of intrauterine fluid accumulation. On the other hand, performing the AI shortly after ovulation slightly increased pregnancy rate per-cycle (48.5% vs. 47%) compared to AI prior to ovulation. A similar effect has previously been reported by others using frozen-thawed semen [11]. More recently, a significant improvement in pregnancy rate of mares inseminated with frozen-thawed semen prior to ovulation was reported compared to those inseminated within 6 h after ovulation [40]. In the latter study, comparable pregnancy rates with those obtained in our study with frozen-thawed semen prior to ovulation were observed (~50%). Pregnancy rate can be further improved if the semen dose is split in two and inseminations are done 12 h apart [41,42], but this was not applied in our study.

The pregnancy rate per-cycle was similar for cycles where oxytocin was applied (43.9%) and cycles where oxytocin was not necessary (50.7%). Since only the mares with intrauterine fluid after insemination were treated with oxytocin, it can be concluded that oxytocin compensates to a certain extent the negative effects of endometritis in susceptible mares. In barren mares, the pregnancy rate per-cycle increased after oxytocin administration. Positive effects of oxytocin alone [18,43] or together with Escherichia coli lipopolysaccharide [44] on pregnancy rate in mares have been previously reported. Barren mares often present an age-associated impaired uterine function which makes them more susceptible for PBIE than other categories [25]. Our results are in accordance with other studies which also showed beneficial effects of PBIE treatment on aged mares [1]. However, to clearly clarify the effect of oxytocin applications on the pregnancy rate in mares with intrauterine fluid, a control group would be necessary.

Cycles in which uterine lavage was required after insemination had a lower chance of pregnancy than cycles in which lavage was not required, but this was not statistically different, probably due to the low number of cases that required lavage. Uterine lavage in mares with PBIE is a widely accepted and commonly performed therapy [7,16,45]. When interpreting the results, it should be considered that uterine lavage was only performed in severe cases of PBIE with fluid accumulations >2 cm in diameter. Other authors also did not observe any detrimental effects of uterine lavage on mare`s fertility, regardless of intrauterine antibiotic infusion post breeding [27,32]. To assess whether uterine lavage is useful as a form of therapy, a control group of endometritis-susceptible mares without lavage would be necessary. It should also be considered that every manipulation of the uterus can lead to bacterial contamination and consecutive inflammation. When low-volume lavage was used for diagnosis of endometritis, up to 11% of the uterine flushes were contaminated, most likely from the vagina [46,47]. Thus, the beneficial effects of uterine lavage on pregnancy rates might be partially hindered by manipulation of the uterus. The increased frequency of required uterine lavage in barren mares was striking, confirming the increased incidence of intrauterine fluid accumulation in older and barren mares [1,9].

## 5. Conclusions

The results of the present study indicate that an insufficiently relaxed cervix at the time of AI together with intrauterine fluid accumulation decrease the chances of conception in mares. Oxytocin administration in mares with intrauterine fluid accumulation leads to satisfactory pregnancy rates, whereas the effect of uterine lavage seems to be limited.

## Data Availability

The data presented in this study are available upon request from the corresponding author.
